# Toward a Personalized Approach in Prebiotics Research

**DOI:** 10.3390/nu9020092

**Published:** 2017-01-26

**Authors:** Moul Dey

**Affiliations:** Department of Health and Nutritional Sciences, Box 2203, South Dakota State University, Brookings, SD 57007, USA; Moul.Dey@sdstate.edu; Tel.: +1-605-688-4050

**Keywords:** prebiotics, disease prevention, dietary bioactives, resistant starch type 4, intervention response variability

## Abstract

Recent characterization of the human microbiome and its influences on health have led to dramatic conceptual shifts in dietary bioactives research. Prebiotic foods that include many dietary fibers and resistant starches are perceived as beneficial for maintaining a healthy gut microbiota. This article brings forward some current perspectives in prebiotic research to discuss why reporting of individual variations in response to interventions will be important to discern suitability of prebiotics as a disease prevention tool.

The ancient Greek physician Hippocrates perceived food as the key player in the maintenance of health, not just as a fuel to run the human body. Since then, scientists have probed deeper into the role of diet in nutrient absorption and bodily function. In the late 1800s, scientists began isolating microorganisms from different parts of the human body including from the digestive tract. Some of these organisms were considered harmful and others beneficial. However, the concept of the human microbiome and its critical role in human health and diseases is more recent, emerging in the 21st century after the advent of next generation sequencing. Mapping microbiome diversity has unlocked many mysteries—but also triggered new questions. The answers to many such questions still elude us, including a very basic but pressing question, “what diet is ideal for a healthy gut microbiome?” It remains unknown if there is an ideal gut microbiome that can be considered “healthy”, nor do we know of one ideal diet that can positively manipulate the microbiome of people of all ages across the globe. Furthermore, a plethora of contradictory research findings on what dietary component may or may not be healthy frequently confuse the public. Not too long ago, dietary fat used to be our worst enemy. With time, that spot was taken over by dietary sugars. Another example is soy, with its many known health benefits and a host of negative side effects [1]. While the reasons behind contradictory nutritional research are multi-faceted, one contributing factor may be researchers designing studies like modern medical research that predominantly aims for disease-specific diagnostic and therapeutic avenues. Scientists prioritize collective outcomes with high statistical significance. While these benchmarks are a sign of a successful clinical trial, individual responses to the dietary treatment are often ignored. For example, recently Zeevi et al. reported widespread and high interpersonal variability in post-prandial glucose response among healthy participants to common dietary components [2]. It is possible that researchers in dietary intervention studies frequently encounter similar variations, but they are under-reported.

Prebiotics are selectively fermented dietary ingredients such as resistant starches and some dietary fibers that change the composition and/or activity of the gastrointestinal microbiota, thus conferring benefits to the host’s health [3]. While this newer type of functional food is increasingly popular, a recent systematic review of six prebiotic trials published before 6 November 2015 suggests that more randomized controlled trials are needed to support their clinical use [4]. More recently our group reported a microbiome signature in response to a resistant starch type 4 (RS4)-enriched diet in individuals with metabolic syndrome (NCT01887964, [5]). It was concluded that RS4 has prebiotic effects with a potential for metabolic disease prevention. This double-blind and placebo-controlled study is among a small number of prebiotic intervention studies conducted under a free-living setting reporting statistically significant changes across microbial composition and abundance, fecal short chain fatty acid levels, and host immunometabolic functions in response to RS4 consumption. More frequently, intervention studies end up being inconclusive or lack statistical power due to wide variability in responses among participants, particularly when conducted within natural living conditions [6]. Interestingly in our study, although statistical significance across most endpoints was observed, response variability was commonplace for bacterial abundance and metabolites as well as clinical endpoints in the host. Relative to average Americans, the study population (Hutterites living in eastern South Dakota) was more genetically homogeneous and had fewer differences in daily lifestyle due to their communal style of living. Their variability in microbiome response, however, came as little surprise. In earlier reports, microbiota varied both in steady state conditions and in response to diet, aging, and other lifestyle changes [7,8,9]. Quite possibly, a deeper mechanistic investigation on how RS4 functions at the molecular level may shed some light on such response variations in the future. However, here the author focusses on one other question that emerges from all of this: will it benefit the scientific community in the long run if such side observations of response variability are routinely reported? Is it possible that we are missing out on information that may hold the key to unlocking some of the mysteries of diet and the microbiome interactions by not reporting individual responses to dietary interventions? Currently, there is little enthusiasm from both scientists and publishers to report such information, as data without statistical significance would rarely contribute to the conclusions drawn from the work. The viewpoint is illustrated in Figure 1. We observed increases in *Ruminococcus lactaris* and *Eubacterium oxidoreducens* in the RS4 group compared to baseline and post-CF (control group, CF) [5]. The most common format for reporting such data is mean % change or fold change along with the *p*-value. Less frequently, individual data points with column means and associated descriptive statistical information are shown (Figure 1B,C). Collective data presentation formats, such as the one shown in Figure 1A, are less helpful in revealing the distinct nature of the two datasets.

Assessing disease risk in susceptible populations remains one major objective of personalized or precision nutrition, allowing for stratifications of subpopulations in a manner that improves the accuracy and cost-effectiveness of interventions and follow-ups. In addition, early prognosis and/or diagnosis may facilitate prophylactic treatment that would otherwise be unsuitable for a larger population. In this context, Zeevi et al. has proposed a machine-learning algorithm approach that integrates multiple features based on a preexisting large cohort data set to predict, for example, glycemic response to real-life meals [2]. While they have reported feasibility, cost-effectiveness of such an approach has not been determined. It must be taken into consideration that no one outcome alone, such as glycemic response, determines the overall health outcome of an individual. For example, being able to predict glycemic response may attenuate the risk of type 2 diabetes, but will not help with the prognosis of hyperlipidemia, heart disease, or cancer. Therefore, many large-scale endeavors, such as those reported by Zeevi et al., will be necessary to predict multiple clinical end-points or intermediate biomarkers before personalized overall health risk determination followed by preventive intervention is possible. For similar reasons, personalized microbiome profiling, while deemed promising as a tool for disease risk stratification, is not ready for translation to a clinical setting. It is only proposed that a predictive microbiome modeling system with more sophisticated readouts integrating multiple aspects of gut microbiota (composition, abundance, metagenomics, meta-transcriptomic, metabolomics, etc.) should be incorporated [10]. In addition, microbiome-based biomarkers for personalized prognostic, diagnostic, and treatment may vary by geographic locations, lifestyle, and many other factors. Therefore, while personalized microbiome profiling may be useful for predicting and mitigating disease, it will take a huge scientific undertaking before it is ready for the clinical setting.

In the past few decades, there has been a surge in metabolic diseases that affect quality of life and pose a substantial medical and economic burden on society. There is a growing interest in preventive measures to modify the risk of metabolic diseases, with diet proposed as a major player in public health promotion. Decades of generalized nutritional recommendations do not seem to be mitigating the metabolic health crisis, although at present there is no alternate to an overall healthy diet and regular physical activity recommendation for long-term health maintenance. Mounting evidence suggests a more personalized approach is required for health promotion through disease prevention and that such personalization cannot entirely rely on human genomic variations in case of complex metabolic diseases. Even taking into account the huge undertaking discussed above, current knowledge about the microbiome suggests that integrating microbiome profiling into patient care will likely allow for a faster, more accurate, and less invasive clinical decision-making processes. In this context, prebiotics will be critical components of personally tailored dietary interventions aimed at altering the microbiome to a more beneficial configuration for disease prevention. While the benefits of dietary fibers, many of which have prebiotic properties, are well-known, their mechanisms of action mostly remain a mystery. Without the knowledge of structure-function relationships between various prebiotics and microbial species as well as further consideration of the bilateral relationship of the microbiome and the host, personalized and effective use of prebiotics for disease prevention will be difficult. Large-scale characterization of the nutrition-microbiome-host metabolism axis will help delineate the integration of prebiotics in personalized diets for prevention of multi-factorial metabolic diseases. One caveat toward such effort is that disease prevention trials typically focus on intermediate outcomes because long-term follow-up of a large enough population, that will both adhere to the intervention as well as provide sufficient statistical power to detect differences, is technically difficult and prohibitively expensive. Regrettably, intermediate outcome measures do not always reflect the true preventive potential of an intervention as reported from the Look AHEAD (Action for Health in Diabetes) trial [11].

Looking to the future, it will be critical to consider the collective effects that are statistically significant, as well as individual response variations, for harnessing the many potential health benefits of prebiotics. Encouraging the scientific community to report variations observed in clinical trials, even if such observations may not meaningfully contribute to the main conclusions of the current study, will be important. Such data may be presented in formats that allow more holistic visualization of study results, including but not limited to, effect sizes, percentile ranking, minimum and maximum values, outliers, means, and medians. A recent commentary in Nature Methods discussed similar data presentation approaches in lieu of sample-to-sample variability and irreproducibility of scientific data, particularly in biomedical disciplines [12]. In addition to that, the author believes, this may also provide an opportunity to build on the vast repertoire of individual response variations that may not otherwise be possible for any one research study to capture. How such data may precisely inform clinical study designs and/or results in the future will depend on effective systematic reviews and meta-analysis outcome from the growing body of such data sets. Nevertheless, the author is hopeful that the information generated will facilitate better predictability of microbial and/or host-physiological response behavior in the direction of early prognosis and prevention.

## Figures and Tables

**Figure 1:**
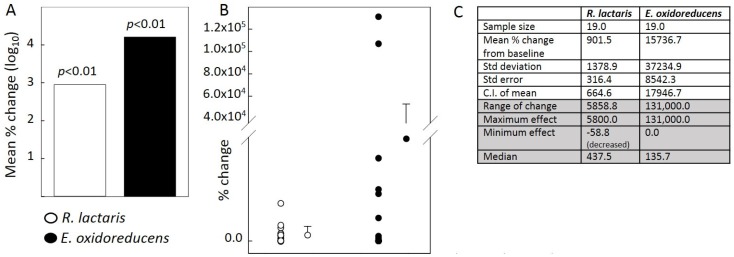
Data presentation formats. (**A**) Relative abundance of two bacterial species before and after RS4 treatment shown as mean % change (log_10_) with corresponding *p* values; (**B**) Percent change from before intervention of the same two bacterial species shown as individual data points with means and standard deviations on the side; (**C**) Descriptive statistical information from the two data sets presented in A and B. The information presented in the last four rows are less frequently reported in clinical trial publications.
